# Para testicular rhabdomyosarcoma in adults: three case reports and review of literature

**DOI:** 10.11604/pamj.2014.19.279.4784

**Published:** 2014-11-14

**Authors:** Lamiae Boudahna, Zineb Benbrahim, Lamiae Amaadour, Aicha Mazouz, Khadija Benhayoune, Yassir Tahiri, Moulay Hassan Farih, Afaf Amarti, Samia Arifi, Nawfel Mellas

**Affiliations:** 1 Medical Oncology Department, Hassan II University Hospital, Fez, Morocco; 2 Laboratory of Pathology, Hassan II University Hospital, Fez, Morocco; 3 Urology department, Hassan II University Hospital, Fez, Morocco

**Keywords:** Para testicular rhabdomyosarcoma, adults, tumor

## Abstract

Paratesticular embryonal rhabdomyosarcoma (RMS) is a rare tumor arising from the mesenchymal tissues of the spermatic cord, epididymis, testis and testicular tunics. We report three cases of adult paratesticular RMS, two embryonic and one pleomorphic rhabdomyosarcoma. All the patients underwent diagnostic orchidectomy. The work up investigations revealed lung metastases. Chemotherapy with Ifosfamide and Doxorubicin was used in two cases, whereas Vincristin- Actinomycin D and Cyclophosphamide was received in one case. An objective partial response was reported in 2 cases, with complete response in one case. Paratesticular RMS is a rare and aggressive tumor. Because of the absence of protocols designed specifically for adult patients, it is necessary to follow therapeutic guidelines in pediatric protocols.

## Introduction

Rhabdomyosarcoma (RMS) is a malignant tumor of mesenchymal origin thought to arise from cells committed to a skeletal muscle lineage. Twenty percent of all cases arise from the genitourinary system [1]. Paratesticular localization includes the epididymis or spermatic cord and occurs mostly in the young people. The first documented case of spermatic cord sarcoma was described by Lesauvage in 1845. Since this date, few cases were reported in the literature especially in adult. The treatment of these cases has evolved over the past decades because of the use of combined modality therapy.

## Patient and observation

### Case 1

**Figure 1:**
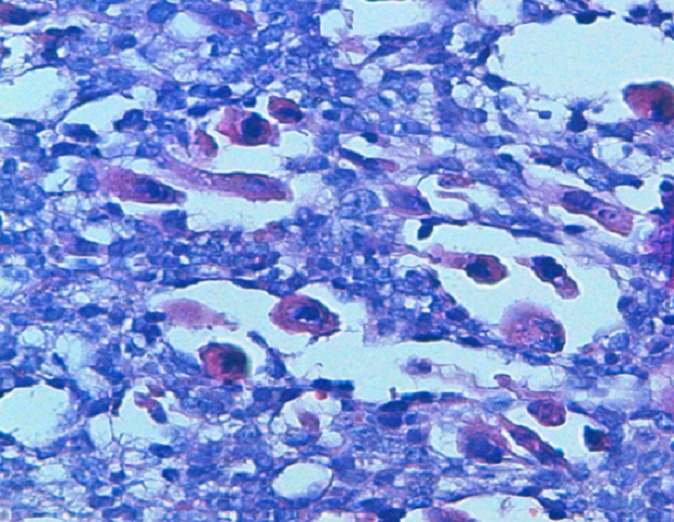
HESx40: rhabdomyoblastic cells at high enlargement

**Figure 2:**
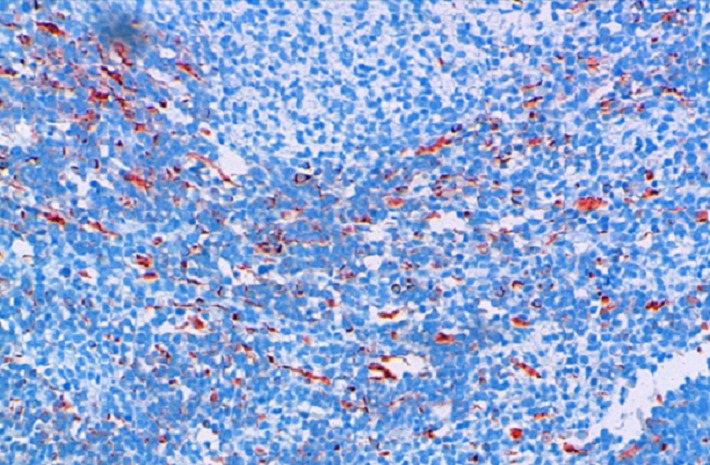
Intense cytoplasmic labeling with anti-desmin antibody

**Figure 3:**
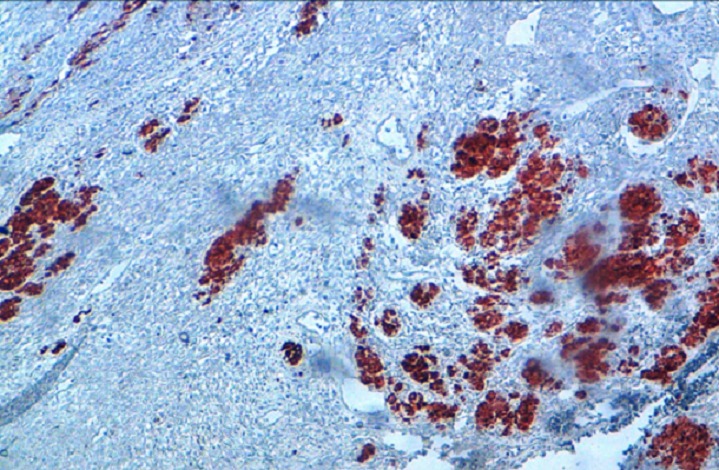
Intense nuclear staining by anti-myogenin antibody

A 18-year-old patient who presented to the department of surgical urology with a painless scrotal mass, he underwent a left inguinal orchidectomy. Histologic examination confirmed an embryonic rhabdomyosarcoma grade III of FNCLCC (Figure 1, Figure 2, Figure 3). The patient presented to our oncology department for further treatment. Tumour markers (testosterone, AFP, HCG, LDH) were normal. A thoraco-abdomino-pelvic CT scan showed one metastasis site in the lung measuring 12 mm. The patient received chemotherapy (Doxorubicin 60mg/m^2^ day 1 and Ifosfamide 1800mg/m^2^ per day from day 1 to day 5 every 21 days) in combination with haematopoietic growth factors for six days. Evaluation after 6 months revealed a full disappearance of the pulmonary metastasis; the patient is monitored quarterly by a CT scan of the chest/abdomen/pelvis.

### Case 2

A 19-year-old patient, presented to our oncology department, with paratesticular embryonic rhabdomyosarcoma diagnosed because a painless scrotal mass. He had undergone initially an orchidectomy. Post operative work up investigations revealed lung and mediastinal lymph nodes metastases. Palliative chemotherapy was received with combined chemotherapy by Ifosfamide and Doxorubicin for six courses. Evaluation showed a full disappearance of pulmonary metastases and stability of mediastinal lymph nodes. Thereafter the patient received radiotherapy for mediastinal lymph nodes and a maintenance chemotherapy with navelbine intravenously associated with oral cyclophosphamide. After 6 months, the patient relapsed in the lung. Actually, a second-line chemotherapy (vincristine 4.5 mg/ m^2^ cyclophosphamide 25 mg / m^2^ orally) is ongoing.

### Case 3

**Figure 4:**
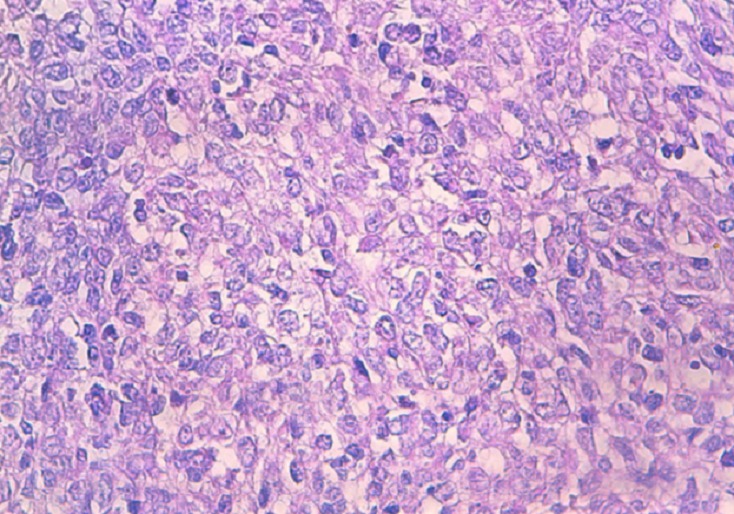
HESx40 the cells are pleomorphic with numerous mitoses

A 25-year-old patient presented to the department of surgical urology with a painless scrotal mass. He underwent a right inguinal orchidectomy. Histologic examination confirmed a pleomorphic RMS (Figure 4). The patient relapsed with locally advanced pelvic mass measuring 11 cm3 months later. Work up staging objectived lung metastases. The patient received chemotherapy with VAC (vinblastine +Actinomycin D +cyclophosfamide) in combination with haematopoietic growth factor for six days. Each chemotherapy session was conducted over five days, with a cycle of 21 days. Evaluation after six courses revealed a major response. Other courses of VAC are ongoing.

## Discussion

Rhabdomyosarcoma (RMS) is the most common soft-tissue sarcoma of childhood. Its incidence is similar in Africo-American and Caucasian and appears to be lower in Asian populations. This histologic subtype develops with two peaks, the first at the age of 4 years and the second at the age of 18 years [2]. Among all cases of rhabdomyosarcoma, approximatively 7% occurs in paratestis. Clinically paratesticular tumour presents as a hard painless inguino-scrotal swelling [3]. A hydrocele can be occasionally present in adults explaining the frequent misdiagnostic of paratesticular rhabdomyosarcoma with hydrocele in this population. In our institution, all patients had a painless scrotal mass. Regarding histologic features, embryonal RMS is predominant and represents 84% of all cases whereas alveolar and spindle cells are less frequent (8% and 5% respectively). In our serie one patient was diagnosed with embryonal RMS and two patients had pleomorphic RMS.

Spread of the tumor is mostly by lymphatics to the iliac and para-aortic nodes, but hematogenous spread does occur most commonly to the lungs and liver [4, 5]. Work up investigations at diagnosis includes physical examination, chest x-ray, bilateral bone marrow smears and biopsies, abdominal and chest computed tomography (CT) scan, and bone scan [6]. Staging of paratesticular rhabdomyosarcoma can be done according to both the tumor-nodes metastases classification and the Intergroup Rhabdomyosarcoma Study system [4]. In the literature, patients are diagnosed at localized stages in 92%. However, all cases in this study had distant metastases at diagnostic. The patients were mostly metastatic in the lungs.

Paratesticular sarcomas are rare. There is no standard treatment. In the localized disease, treatment strategies include radical high inguinal orchidectomy, retroperitoneal lymph node dissection, chemotherapy and radiotherapy [7–9]. Some authors recommend ipsilateral nerve-sparing retroperitoneal lymph node dissection (RPLND) for all boys 10 years of age or older. This therapeutic approach is based upon results from the Intergroup Rhabdomyosarcoma Study IV which concluded that three-year PFS in boys over the age of 10 who had apparently localized paratesticular RMS but did not undergo routine RPLND was significantly worse than that of younger boys (68 versus 90 percent). Besides, the histologic confirmation of nodal metastases is helpful for decision making since patients with positive nodes are referred for postoperative RT as well as adjuvant chemotherapy. An alternative approach for patients with clinically enlarged retroperitoneal nodes is the administration of an adjuvant chemotherapy regimen (VAC or vincristine plus dactinomycin and ifosfamide (VAI)). The development of this adjuvant therapy has increased survival in patients with localized disease to approximately 60% [10]. In the metastatic setting, many protocols of chemotherapy have been tried. VAC, IVA, and VIE protocols (V: vincristine, A: actinomycin, I: ifosfamide, E: etoposide, and C: cyclophosphamide) and better results were observed with VAC protocol [11–13]. In our serie two cases received chemotherapy by MAI, and one patient was treated by VAC. The role of whole-lung RT (generally to 14.4 Gy) for patients with overt pulmonary metastases is not consensual; some protocols recommend it given the radiosensitivity of RMS. The prognosis of paratesticular rhabdomyosarcoma is extremely poor. Patients in the Intergroup Rhabdomyosarcoma Study IV had a 5-year survival rate of 22.2%. Furthermore, age seems to be a prognostic factor with a worse prognosis in adult patients than children (with a 5-year event-free survival and 5-year overall survival of 28% and 40%, respectively [4].

## Conclusion

Paratesticular RMS is a rare and aggressive tumor. In the absence of protocols designed specifically for adult patients, it is necessary to follow therapeutic guidelines in pediatric protocols. Systemic chemotherapy is essential in both early and advanced disease and has resulted in improved survival outcomes.
